# Ubiquitylomes Analysis of the Whole blood in Postmenopausal Osteoporosis Patients and healthy Postmenopausal Women

**DOI:** 10.1111/os.12556

**Published:** 2019-11-25

**Authors:** Yi‐ran Yang, Chun‐wen Li, Jun‐hua Wang, Xiao‐sheng Huang, Yi‐feng Yuan, Jiong Hu, Kang Liu, Bo‐cheng Liang, Zhong Liu, Xiao‐lin Shi

**Affiliations:** ^1^ The Second Clinical Medical College, Zhejiang Chinese Medical University Hangzhou China; ^2^ Department of Diagnostics of Traditional Chinese Medicine, College of Basic Medical Science Zhejiang Chinese Medical University Hangzhou China; ^3^ The Second Affiliated Hospital Zhejiang Chinese Medical University Hangzhou China

**Keywords:** Postmenopausal osteoporosis, Proteome, Ubiquitylome

## Abstract

**Objectives:**

To determine the mechanisms of ubiquitination in postmenopausal osteoporosis and investigate the ubiquitinated spectrum of novel targets between healthy postmenopausal women and postmenopausal osteoporosis patients, we performed ubiquitylome analysis of the whole blood of postmenopausal women and postmenopausal osteoporosis patients.

**Methods:**

To obtain a more comprehensive understanding of the postmenopausal osteoporosis mechanism, we performed a quantitative assessment of the ubiquitylome in whole blood from seven healthy postmenopausal women and seven postmenopausal osteoporosis patients using high‐performance liquid chromatography fractionation, affinity enrichment, and liquid chromatography coupled to tandem mass spectrometry (LC‐MS/MS). To examine the ubiquitylome data, we performed enrichment analysis using an ubiquitylated amino acid motif, Gene Ontology (GO) and the Kyoto Encyclopedia of Genes and Genomes (KEGG) pathway.

**Results:**

Altogether, 133 ubiquitinated sites and 102 proteins were quantified. A difference of more than 1.2 times is considered significant upregulation and less than 0.83 significant downregulation; 32 ubiquitinated sites on 25 proteins were upregulated and 101 ubiquitinated sites on 77 proteins were downregulated. These quantified proteins, both with differently ubiquitinated sites, participated in various cellular processes, such as cellular processes, biological regulation processes, response to stimulus processes, single‐organism and metabolic processes. Ubiquitin conjugating enzyme activity and ubiquitin‐like protein conjugating enzyme activity were the most highly enriched in molecular function of upregulated sites with corresponding proteins, but they were not enriched in downregulated in sites with corresponding proteins. The KEGG pathways analysis of quantified proteins with differentiated ubiquitinated sites found 13 kinds of molecular interactions and functional pathways, such as glyoxylate and decarboxylate metabolism, dopaminergic synapse, ubiquitin‐mediated proteolysis, salivary secretion, coagulation and complement cascades, Parkinson's disease, and hippo signaling pathway. In addition, hsa04120 ubiquitin‐mediated proteolysis was the most highly enriched in proteins with upregulated sites, hsa04610 complement and coagulation cascades was the most highly enriched in proteins with downregulated ubiquitinated sites, and hsa04114 Oocyte meiosis was the most highly enriched among all differential proteins.

**Conclusion:**

Our study expands the understanding of the spectrum of novel targets that are differentially ubiquitinated in whole blood from healthy postmenopausal women and postmenopausal osteoporosis patients. The findings will contribute toward our understanding of the underlying proteostasis pathways in postmenopausal osteoporosis and the potential identification of diagnostic biomarkers in whole blood.

## Introduction

The metabolism of bone cells is strongly regulated by estrogens and, therefore, postmenopausal osteoporosis is the most typical form of osteoporosis, which is characterized by low bone mass and microstructure damage of the bone tissue, leading to increased bone fragility and the risk of fracture^1^. Osteoporosis causes huge economic losses worldwide. Approximately 10 million men and women in the USA have osteoporosis^2^, 75 million people in Europe, North America and Japan are affected by it^3^, while the prevalence of osteoporosis in central south Chinese postmenopausal women is approximately 39.4%^4^.

Although the role of estrogen on bone metabolism has been documented, the mechanism of postmenopausal osteoporosis remains unclear and diagnostic strategies for postmenopausal osteoporosis are lacking.

Over the past century, many types of histone post‐translational modifications (PTM) have been identified, including lysine acetylation, arginine and lysine methylation, phosphorylation, proline isomerization, ubiquitination (Ub), ADP ribosylation, arginine citrullination, SUMOylation, carbonylation, and biotinylation^5^, ^6^. It is known that the bone remodeling cycle is a balanced process that depends on the interaction, differentiation, and functions of the mesenchymal osteoblastic lineage and the hematopoietic osteoplastic lineage to maintain homeostasis of bone mass. Many histone post‐translational modifications are involved in bone remodeling cycle regulation.

Works such as Hein *et al*.^7^ and Li *et al*.^8^ have established that advanced glycation end products (AGE) could biphasically modulate bone resorption in osteoclast‐like cells and modify proteins in osteoporotic bone. A phosphoproteome of Milani *et al*.^9^ revealed that phosphorylation is extremely important during osteoblast adhesion. In addition, a phosphoproteome study by Marumoto *et al*.^10^ further reveals that hedgehog signaling has a critical role in osteoblast morphological transitions. However, the review of Bradley *et al*.^11^ demonstrates that histone deacetylases have emerged as crucial regulators of both intramembranous and endochondral bone formation.

In summary, their results indirectly demonstrate the feasibility of revealing the mechanism of bone remodeling through the histone post‐translational modifications. The research of Zhang *et al*.^12^ provides further relevant evidence. Zhang *et al*. examined the dynamics of distinct histone modifications during osteogenesis, including the dynamics of: H3K9/K14 and H4K12 acetylation; H3K4 mono‐, di‐ and tri‐methylation; H3K9 di‐methylation and H3K27 tri‐methylation in osteogenic genes, runt‐related transcription factor 2 (Runx2), osterix (Osx), alkaline phosphatase, and bone sialoprotein and osteocalcin during C3H10T1/2 osteogenesis.

However, the most important post‐translational modifications are ubiquitin modifications in bone metabolism. The ubiquitin‐proteasome system is one of the major quality control pathways responsible for cellular homeostasis^13^, ^14^, ^15^.

Ubiquitination is a posttranslational modification of proteins that controls almost every cellular metabolic pathway through a variety of combinations of linkages, either as a single moiety or as polymers^16^. These cellular metabolic pathways include, but are not limited to, transcription, cell signaling^17^, endocytic trafficking, DNA damage and repair^18^, and cell‐cycle control^19^. The ubiquitin system in humans consists of 2 E1, 35 E2, more than 600 E3 ubiquitin ligases, and hundreds of deubiquitylases^20^.

For more than a decade, investigators have shown that ubiquitin‐proteasome‐mediated protein degradation is critical in regulating the balance between bone formation and bone resorption^21^.

In addition, several studies have found that many ubiquitinases play an important role in bone metabolism, such as poly‐ubiquitination‐mediated RUNX2 degradation, which is an important cause of bone loss^22^, ^23^. Surveys such as that conducted by Zhou *et al*.^24^ have shown that RNF185 negatively regulates osteogenesis through the degradation of Dvl2 and the downregulation of the canonical Wnt signaling pathway. Many studies indicate that the muscle‐specific RING‐finger1 (MuRF1), a muscle‐specific ubiquitin ligas, is involved in osteoblastic bone formation and osteoclastic bone resorption^25^, ^26^, ^27^. Xu *et al*. 28 established that SMURF2 is an important regulator of the critical communication between osteoblasts and osteoclasts; the bone mass phenotype in Smurf2‐deficient and Smurf1‐deficient mice is opposite, indicating that SMURF2 has a non‐overlapping and opposite function to SMURF1. Jin *et al*.^29^ found that Bre promoted the Mdm2‐mediated p53 ubiquitination and degradation by physically interacting with p53, and that Bre has a novel function in osteoblast differentiation through modulating the stability of p53.

In contrast, quantitative analysis of ubiquitylomes has proven to be a valuable tool for elucidating targets and mechanisms of the ubiquitin signaling systems^13^, ^14^, ^15^, as well as gaining insights into many diseases^30^, such as neuroblastoma^31^, cancer^32^, and Alzheimer's disease^33^.

To date, although the importance of ubiquitin modifications in bone metabolism has been reported, few studies have investigated ubiquitylomes in postmenopausal osteoporosis.

Here, we used ubiquitylomes analysis of the whole blood of postmenopausal women and postmenopausal osteoporosis patients, to discover the mechanism of ubiquitination in postmenopausal osteoporosis and investigate the ubiquitinated spectrum of novel targets between healthy postmenopausal women and postmenopausal osteoporosis patients. This expanded our understanding of the spectrum of novel targets that are differentially ubiquitinated in whole blood from healthy postmenopausal women and postmenopausal osteoporosis patients. Overall, the study highlights the utility of liquid chromatography coupled to tandem mass spectrometry (LC‐MS/MS) in performing comprehensive mapping of the human blood ubiquitylome changes in postmenopausal osteoporosis, which provides insight into underlying proteostasis pathways and targets that may have potential as novel diagnostic biomarkers in blood.

## Materials and Methods

### *Human Subjects*

This study included seven consecutive, unrelated, late postmenopausal women with osteoporosis and seven healthy postmenopausal women who visited the Second Affiliated Hospital of Zhejiang Chinese Medicine University. All subjects were Chinese Han females. Postmenopausal status was defined as no menses for at least 1 year after their last menses. Bone mineral density (BMD) of the lumbar spine (L_1_–L_4_) and femoral neck was measured by dual‐energy X‐ray absorptiometry using a QDR‐4500w instrument (Hologic, USA) at the Second Affiliated Hospital of Zhejiang Chinese Medicine University. The average age of the osteoporosis patients was 66.1 ± 3.4 years, with a range of 54–81 years. Seven age‐matched postmenopausal healthy volunteers (average age 68.9 ± 2.5 years, with a range of 58–79 years) were also recruited in Hangzhou.

### *Diagnostic Criteria*

The diagnosis of osteoporosis was based on the criteria recommended by the World Health Organization^34^, which included that the bone density (g/cm^2^) of the lumbar vertebra normal position and femoral neck was surveyed using dual‐energy X‐ray absorptiometry, compared with a normal adult of the same gender and race; T ≤ −2.5 could be diagnosed as osteoporosis, where T = (the standard deviation of measured value − peak bone mass)/normal adult bone density.

### *Inclusion Criteria*

Patients were included in the study based on the following criteria: (i) they conformed to the osteoporosis diagnostic criteria; (ii) they were postmenopausal women; and (iii) they were between 50 and 89 years old.

### *Exclusion Criteria*

Patients were excluded from the study based on the following criteria, which are derived from a previous study^35^: (i) all individuals with disorders known to cause abnormalities in the metabolism of bone or calcium, such as diabetes, Cushing's syndrome, functional change of the thyroid or parathyroid, osteomalacia, rheumatoid arthritis, multiple myeloma, bone tumor, osteoarthrosis, Paget's disease, and osteogenesis imperfecta; (ii) the individuals that also had severe primary cardiac diseases, or diseases of the cerebral vessels or hematopoietic system; (iii) the individuals that also had severe liver function or renal insufficiencies; (iv) the individuals who had taken drugs within the past 6 months that affect bone metabolism, such as estrogen, steroid hormones, calcitonin, parathyroid hormones, bisphosphonates, fluoride, vitamin D, anticonvulsant drugs, and diuretics; (v) the individuals who had a medical history of mental illness; and (vi) the individuals who had Alzheimer's disease.

### *Ethical Review*

The study protocol was approved by the local Ethics Committee of the Second Affiliated Hospital of Zhejiang Chinese Medicine University and informed consent was obtained from all subjects.

### *Trypsin Enzymatic Hydrolysis*

We added 8 mol urea to the sample to adjust the volume, then added dithiothreitol to a final concentration of 5 mmol, reducing at 56 °C for 30 min. Iodoacetamide was then added to a final concentration of 11 mmol and incubated for 15 min at room temperature in the dark. Finally, the urea concentration of the sample was diluted to less than 2 mol. Trypsin was added at a mass ratio of 1:50 (pancreatin: protein) and digested overnight at 37 °C. Trypsin was added at a mass ratio of 1:100 (pancreatin: protein) and continued to digest for 4 h.

### *High‐Performance Liquid Chromatography Fractionation*

The peptides were fractionated by high pH reverse phase high‐performance liquid chromatography and the column was Agilent 300 Extend C18 (5 μm particle size, 4.6 mm id, 250 mm long). Finally, the fractional gradient of the peptide was 8%–32% acetonitrile, pH 6.0, allowing 60 min time to separate 60 components, and then the peptides were combined into four components, and the combined components were vacuum freeze‐dried for subsequent operations.

### *Affinity Enrichment*

The peptide was dissolved in IP buffer solution (100 mmol NaCl, 1 mmol EDTA, 50 mmol Tris‐HCl, 0.5% NP‐40, pH 8.0), and the supernatant was transferred to the pre‐washed ubiquitinated resin (resin number) PTM‐1104, from Hangzhou Jingjie Biotechnology, PTM Bio, placed on a rotary shaker at 4°C, gently shaken, and incubated overnight. Then, the resin was washed four times with IP buffer solution and twice with deionized water. Finally, the resin‐bound peptide was 0.1% trifluoroacetic acid eluate, eluted three times in total, and the eluate was collected and vacuum‐dried and drained. The salt was removed according to the C18 ZipTips instructions, vacuum‐dried, and drained for liquid‐mass spectrometry analysis.

### *LC‐MS/MS Analysis*

The tryptic peptides were dissolved in phase A in an aqueous solution of 0.1% formic acid and 2% acetonitrile; buffer B was an aqueous solution of 0.1% formic acid and 90% acetonitrile. Liquid phase gradient setting: 0–26 min, 5%–22% B; 26–34 min, 22%–35% B; 34–37 min, 35%–80% B; 37–40 min, 80% B, flow rate maintenance at 350 nL/min. The peptides were separated using an ultra‐high‐performance liquid phase system and injected into the NSI ion source for ionization, and then analyzed by Orbitrap FusionTM (Thermo) mass spectrometry. The ion source voltage was 2.0 kV, and the peptide precursor and its secondary fragments were detected and analyzed by high‐resolution Orbitrap. The primary mass spectrometer scan range was 350–1550 m/z and the scan resolution was set to 60 000; the secondary mass spectrometry scan range was 100 m/z and the Orbitrap scan resolution was 15 000. After the first‐stage scanning, the first 20 peptides with the highest signals were selected to enter the HCD collision cell and 35% of the fragmentation energy was used for secondary mass spectrometry. Finally, the automatic gain control (AGC) was set to 5E4, the signal threshold to 5000 ions/s, the maximum injection time to 200 ms, and the dynamic exclusion time of the tandem mass spectrometry to 15 s to avoid the parent ion. Then the scan was repeated.

### *Database Search*

Secondary mass spectral data was retrieved using Maxquant (v1.5.2.8). Search parameter settings: The database used was SwissProthuman (20 203 sequences), the anti‐library was added to calculate the false positive rate (FDR) caused by random matching, and a common pollution database was added to the database to eliminate the effect of contaminated proteins on the results. The enzyme digestion mode was Trypsin/P; the number of missed sites was 2; the minimum length of the peptide was seven amino acid residues; the maximum number of peptides was five; First search range was set to 5 ppm for precursor ions. Main search range set to 5 ppm and 0.02 Da for fragment ions. The mass error tolerance was 20 and 5 ppm. Finally, the fixed modification was cysteine alkylation, Author: the variable modification was methionine oxidation, protein N‐terminal acetylation, and lysine ubiquitination. The FDR for protein identification and PSM identification was 1%.

### *Bioinformatics Analysis*

The Gene Ontology (GO) annotation proteome was derived from the UniProt‐GOA database (http://www.ebi.ac.uk/GOA/) and the InterProScan software^36^, ^37^. First, identified protein ID was converted to UniProt ID and then mapped to GO IDs by protein ID. If some identified proteins were not annotated by the UniProt‐GOA database, the InterPro Scan software would be used to annotate proteins’ GO functional classification based on the protein sequence alignment method. Finally, proteins are classified by GO annotation based on three categories: biological process, cellular component, and molecular function.

We used the Kyoto Encyclopedia of Genes and Genomes (KEGG) database for protein pathway analysis^38^. First, we used the KEGG online service tool KAAS to annotate proteins’ KEGG database description. Then, we mapped the annotation result on the KEGG pathway database using the KEGG online service tool KEGG mapper. The protein complex analysis was conducted using the CORUM protein complex database. We used the InterPro domain database for protein domain annotation and Motif‐x for protein analysis.

## Results

### *Overview of Global Ubiquitylome upon Postmenopausal Osteoporosis*

Altogether, 133 ubiquitinated sites and 102 proteins were quantified. The analysis of ubiquitylomes the whole blood in seven healthy postmenopausal women and seven postmenopausal osteoporosis patients revealed that 32 ubiquitinated sites on 25 proteins were upregulated and 101 ubiquitinated sites on 77 proteins were downregulated. The difference is more than 1.2 times as significant upregulation and less than 0.83 as a significant downregulation (*P* < 0.05, Table 1). Here, there are multiple ubiquitination sites on the same protein, some of which are rising and some are different.

**Table 1:** The differentially expressed ubiquitinated sites and proteins in ubiquitylome of postmenopausal osteoporosis patients and healthy postmenopausal women

Protein accession	Ratio	Regulated type	Protein description	Gene name	Score
P02656	0.212	Down	Apolipoprotein C‐III	APOC3	161.24
O43765	0.564	Down	Small glutamine‐rich tetratricopeptide repeat‐containing protein alpha	SGTA	131.66
P00918	0.608	Down	Carbonic anhydrase 2	CA2	125.73
Q04323	0.26	Down	UBX domain‐containing protein 1	UBXN1	88.708
Q9P107	13.569	Up	GEM‐interacting protein	GMIP	50.314
P02675	0.373	Down	Fibrinogen beta chain	FGB	117.84
P02675	0.192	Down	Fibrinogen beta chain	FGB	132.81
P00736	0.115	Down	Complement C1r subcomponent	C1R	70.165
P21980	0.595	Down	Protein‐glutamine gamma‐glutamyltransferase 2	TGM2	102.53
Q9BQE3	0.276	Down	Tubulin alpha‐1C chain	TUBA1C	137.18
Q9BQE3	0.546	Down	Tubulin alpha‐1C chain	TUBA1C	115.54
Q9BQE3	0.611	Down	Tubulin alpha‐1C chain	TUBA1C	70.783
Q9BQE3	0.416	Down	Tubulin alpha‐1C chain	TUBA1C	95.618
Q9BQE3	0.122	Down	Tubulin alpha‐1C chain	TUBA1C	119.39
P10599	0.336	Down	Thioredoxin	TXN	92.19
P02774	0.121	Down	Vitamin D‐binding protein	GC	50.786
P15153	0.09	Down	Ras‐related C3 botulinum toxin substrate 2	RAC2	50.108
P02647	0.483	Down	Apolipoprotein A‐I	APOA1	247.38
P28066	0.23	Down	Proteasome subunit alpha type‐5	PSMA5	161.68
P32119	26.6	Up	Peroxiredoxin‐2	PRDX2	92.039
P32119	0.696	Down	Peroxiredoxin‐2	PRDX2	145.46
Q13228	5.732	Up	Selenium‐binding protein 1	SELENBP1	141.07
P63104	0.564	Down	14‐3‐3 protein zeta/delta	YWHAZ	199.8
P16157	0.589	Down	Ankyrin‐1	ANK1	150.11
P16157	0.525	Down	Ankyrin‐1	ANK1	220.63
P16157	0.184	Down	Ankyrin‐1	ANK1	154.72
P16157	0.384	Down	Ankyrin‐1	ANK1	271.08
P16157	2.199	Up	Ankyrin‐1	ANK1	122.28
P16157	1.482	Up	Ankyrin‐1	ANK1	239.87
P02649	0.183	Down	Apolipoprotein E	APOE	160.18
Q14687	0.598	Down	Genetic suppressor element 1	GSE1	116.54
P20618	0.416	Down	Proteasome subunit beta type‐1	PSMB1	132.13
P02671	0.805	Down	Fibrinogen alpha chain	FGA	81.017
P02671	0.554	Down	Fibrinogen alpha chain	FGA	71.153
P04040	2.279	Up	Catalase	CAT	109.83
P00441	0.442	Down	Superoxide dismutase [Cu‐Zn]	SOD1	80.96
Q9C0C9	1.597	Up	(E3‐independent) E2 ubiquitin‐conjugating enzyme	UBE2O	112.26
Q9C0C9	4.967	Up	(E3‐independent) E2 ubiquitin‐conjugating enzyme	UBE2O	68.536
Q9C0C9	0.581	Down	(E3‐independent) E2 ubiquitin‐conjugating enzyme	UBE2O	97.597
Q9C0C9	4.099	Up	(E3‐independent) E2 ubiquitin‐conjugating enzyme	UBE2O	88.021
Q9C0C9	0.809	Down	(E3‐independent) E2 ubiquitin‐conjugating enzyme	UBE2O	124.21
P62258	0.227	Down	14–3‐3 protein epsilon	YWHAE	76.847
P25789	0.473	Down	Proteasome subunit alpha type‐4	PSMA4	105.2
P25789	0.349	Down	Proteasome subunit alpha type‐4	PSMA4	127.56
P27348	0.36	Down	14‐3‐3 protein theta	YWHAQ	120.46
P22314	95.567	Up	Ubiquitin‐like modifier‐activating enzyme 1	UBA1	168.62
P22314	0.166	Down	Ubiquitin‐like modifier‐activating enzyme 1	UBA1	118.32
P28070	0.489	Down	Proteasome subunit beta type‐4	PSMB4	205.62
P23634	0.405	Down	Plasma membrane calcium‐transporting ATPase 4	ATP2B4	85.355
P0C0L4	0.43	Down	Complement C4‐A	C4A	109.71
P62158	12.941	Up	Calmodulin	CALM1	62.099
P30153	1.362	Up	Serine/threonine‐protein phosphatase 2A 65 kDa regulatory subunit A alpha isoform	PPP2R1A	120.03
Q96BN8	0.071	Down	Ubiquitin thioesterase otulin	OTULIN	109.07
P11171	0.457	Down	Protein 4.1	EPB41	184.24
P11171	2.742	Up	Protein 4.1	EPB41	63.691
P11171	0.416	Down	Protein 4.1	EPB41	125.97
P11171	0.266	Down	Protein 4.1	EPB41	54.772
P67775	0.352	Down	Serine/threonine‐protein phosphatase 2A catalytic subunit alpha isoform	PPP2CA	78.149
P02042	1.625	Up	Hemoglobin subunit delta	HBD	120.23
P68036	1.742	Up	Ubiquitin‐conjugating enzyme E2 L3	UBE2L3	90.793
P16452	1.313	Up	Erythrocyte membrane protein band 4.2	EPB42	360.48
P16452	0.72	Down	Erythrocyte membrane protein band 4.2	EPB42	189.43
P02652	0.223	Down	Apolipoprotein A‐II	APOA2	89.123
P02652	0.568	Down	Apolipoprotein A‐II	APOA2	124.12
P02765	0.573	Down	Alpha‐2‐HS‐glycoprotein	AHSG	134.99
P40925	4.17	Up	Malate dehydrogenase, cytoplasmic	MDH1	95.531
P54727	0.629	Down	UV excision repair protein RAD23 homolog B	RAD23B	167.89
P54252	0.464	Down	Ataxin‐3	ATXN3	72.315
P05198	0.564	Down	Eukaryotic translation initiation factor 2 subunit 1	EIF2S1	103.02
Q04917	0.154	Down	14–3‐3 protein eta	YWHAH	54.259
P00491	1.549	Up	Purine nucleoside phosphorylase	PNP	140.96
Q13875	2.37	Up	Myelin‐associated oligodendrocyte basic protein	MOBP	73.233
Q13875	2.37	Up	Myelin‐associated oligodendrocyte basic protein	MOBP	73.233
P01857	0.489	Down	Ig gamma‐1 chain C region	IGHG1	156.7
P27169	0.147	Down	Serum paraoxonase/arylesterase 1	PON1	55.064
O15554	1.412	Up	Intermediate conductance calcium‐activated potassium channel protein 4	KCNN4	47.039
P05154	0.405	Down	Plasma serine protease inhibitor	SERPINA5	119.68
Q5T4S7	0.751	Down	E3 ubiquitin‐protein ligase UBR4	UBR4	98.175
Q5T4S7	0.263	Down	E3 ubiquitin‐protein ligase UBR4	UBR4	75.764
P25786	0.528	Down	Proteasome subunit alpha type‐1	PSMA1	169.89
P49720	0.326	Down	Proteasome subunit beta type‐3	PSMB3	190.26
Q58WW2	0.464	Down	DDB1‐ and CUL4‐associated factor 6	DCAF6	121.61
Q58WW2	0.327	Down	DDB1‐ and CUL4‐associated factor 6	DCAF6	147.4
P02549	1.491	Up	Spectrin alpha chain, erythrocytic 1	SPTA1	156.67
P02549	4.173	Up	Spectrin alpha chain, erythrocytic 1	SPTA1	124.08
P02549	0.779	Down	Spectrin alpha chain, erythrocytic 1	SPTA1	155.66
P02549	0.624	Down	Spectrin alpha chain, erythrocytic 1	SPTA1	156.36
P02549	0.687	Down	Spectrin alpha chain, erythrocytic 1	SPTA1	228.19
P02549	0.301	Down	Spectrin alpha chain, erythrocytic 1	SPTA1	158.29
P61088	2.626	Up	Ubiquitin‐conjugating enzyme E2 N	UBE2N	190.41
P54725	0.532	Down	UV excision repair protein RAD23 homolog A	RAD23A	168.59
Q00610	0.208	Down	Clathrin heavy chain 1	CLTC	65.231
P29144	0.572	Down	Tripeptidyl‐peptidase 2	TPP2	104.18
P51811	0.666	Down	Membrane transport protein XK	XK	138.89
Q9BSL1	9.707	Up	Ubiquitin‐associated domain‐containing protein 1	UBAC1	180.88
P04075	0.53	Down	Fructose‐bisphosphate aldolase A	ALDOA	139.47
P02743	0.338	Down	Serum amyloid P‐component	APCS	121.99
Q16531	10.591	Up	DNA damage‐binding protein 1	DDB1	123.63
Q5VW32	0.264	Down	BRO1 domain‐containing protein BROX	BROX	57.532
P30043	0.511	Down	Flavin reductase (NADPH)	BLVRB	187.78
P62987	0.595	Down	Ubiquitin‐60S ribosomal protein L40	UBA52	136.96
P45974	0.742	Down	Ubiquitin carboxyl‐terminal hydrolase 5	USP5	173.72
P02655	0.238	Down	Apolipoprotein C‐II	APOC2	80.585
O14818	0.484	Down	Proteasome subunit alpha type‐7	PSMA7	56.916
Q15843	9.91	Up	NEDD8	NEDD8	206.18
Q15843	3.159	Up	NEDD8	NEDD8	207.82
Q15843	13.049	Up	NEDD8	NEDD8	84.365
P13716	0.402	Down	Delta‐aminolevulinic acid dehydratase	ALAD	53.453
Q9NR09	0.242	Down	Baculoviral IAP repeat‐containing protein 6	BIRC6	45.357
P0CG05	0.383	Down	Ig lambda‐2 chain C regions	IGLC2	131.82
Q96GG9	0.645	Down	DCN1‐like protein 1	DCUN1D1	120.76
P55072	0.18	Down	Transitional endoplasmic reticulum ATPase	VCP	45.883
P55072	0.622	Down	Transitional endoplasmic reticulum ATPase	VCP	94.114
P01009	0.13	Down	Alpha‐1‐antitrypsin	SERPINA1	71.451
P02679	0.305	Down	Fibrinogen gamma chain	FGG	123.86
Q93034	2.055	Up	Cullin‐5	CUL5	211.64
P07738	0.593	Down	Bisphosphoglycerate mutase	BPGM	134.44
P23526	13.226	Up	Adenosylhomocysteinase	AHCY	49.188
P04406	0.66	Down	Glyceraldehyde‐3‐phosphate dehydrogenase	GAPDH	92.439
P27105	0.51	Down	Erythrocyte band 7 integral membrane protein	STOM	134.73
P00915	0.322	Down	Carbonic anhydrase 1	CA1	65.428
P00915	0.705	Down	Carbonic anhydrase 1	CA1	147.17
P00915	0.116	Down	Carbonic anhydrase 1	CA1	110.34
P68371	0.547	Down	Tubulin beta‐4B chain	TUBB4B	167.61
P28289	0.655	Down	Tropomodulin‐1	TMOD1	95.822
P69905	0.717	Down	Hemoglobin subunit alpha	HBA1	199.81
P69905	3.223	Up	Hemoglobin subunit alpha	HBA1	168.55
P11277	0.748	Down	Spectrin beta chain, erythrocytic	SPTB	108.63
P11277	0.47	Down	Spectrin beta chain, erythrocytic	SPTB	142.89
P11166	0.387	Down	Solute carrier family 2, facilitated glucose transporter member 1	SLC2A1	136.57
Q8IZP2	0.627	Down	Putative protein FAM10A4	ST13P4	127.36
Q99436	0.099	Down	Proteasome subunit beta type‐7	PSMB7	103.88
Q99436	0.17	Down	Proteasome subunit beta type‐7	PSMB7	103.88

### *Gene Ontology of Differentially Quantified Proteins*

**Figure 1:**
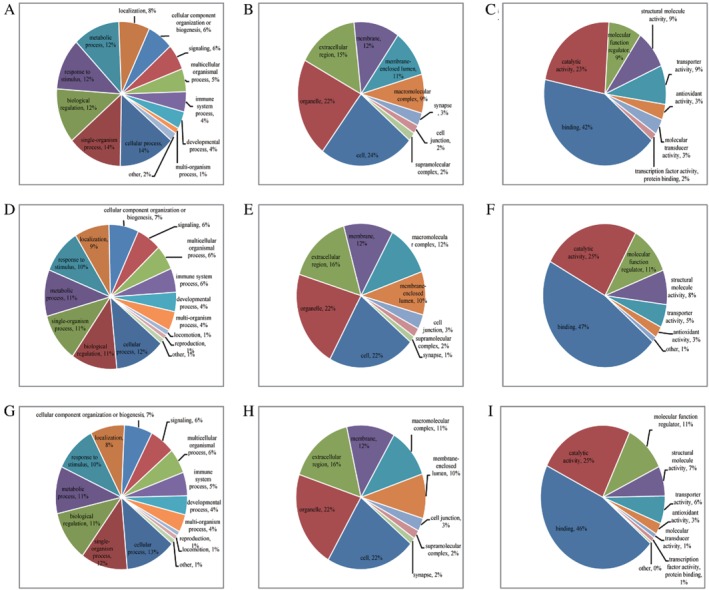
Gene Ontology annotations of quantified proteins with differential sites: (A) biological process annotation of quantified proteins with upregulated ubiquitinated sites; (B) cellular component annotation of quantified proteins with upregulated ubiquitinated sites; (C) molecular function annotation of quantified proteins with upregulated ubiquitinated sites; (D) biological process annotation of quantified proteins with downregulation ubiquitinated sites; (E) cellular component annotation of quantified proteins with downregulation ubiquitinated sites; (F) molecular function annotation of quantified proteins with downregulation ubiquitinated sites; (G) biological process annotation of all quantified proteins; (H) cellular component annotation of all quantified proteins; and (I) molecular function annotation of all quantified proteins.

To investigate the features of the whole blood differentially expressed proteins upon postmenopausal osteoporosis patients and healthy postmenopausal women, classification of GO annotation was performed. GO is an important bioinformatics analysis method and tool for expressing various properties of genes and gene products. It is divided into three broad categories: biological process, cellular component, and molecular function. In quantified proteins with upregulated ubiquitinated sites, cellular process (14%), single‐organism process (14%), biological regulation process (12%), response to stimulus process (12%), and metabolic process (12%) were leading categories when biological processes were evaluated (Fig. 1A); cell (24%) and organelle (22%)‐related proteins stood out in the cellular component analysis (Fig. 1B); molecular function analysis showed that the top two functions were binding (42%) and catalytic activity (23%, Fig. 1C).

As for quantified proteins with downregulation ubiquitinated sites, cellular process (12%), biological regulation process (11%), single‐organism process (11%), metabolic process (11%), response to stimulus process (10%) and localization process (9%) were important in biological processes (Fig. 1D); cell (22%) and organelle (22%) related proteins still stood in leadership in the cellular component analysis (Fig. 1E); the top two functions of molecular function were binding (47%) and catalytic activity (25%, Fig. 1F).

**Figure 2:**
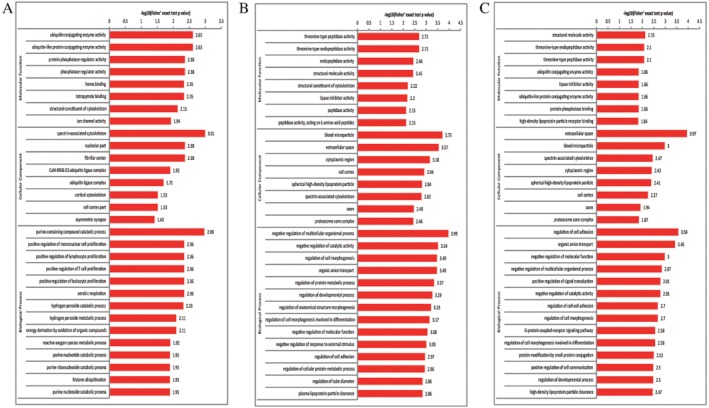
Gene Ontology enrichment results for upregulated ubiquitinated sites with corresponding proteins (A), downregulated ubiquitinated sites with corresponding proteins (B), and all differentially expressed proteins (C), the horizontal axis value is a negative logarithmic transformation of significant *P* values (*P* < 0.05).

To explore the cellular functions of differentially regulated proteins in whole blood from healthy postmenopausal women and postmenopausal osteoporosis patients, functional enrichment was conducted in the GO pathway. In the GO functional clustering analysis of upregulated sites with corresponding proteins, ubiquitin conjugating enzyme activity and ubiquitin‐like protein conjugating enzyme activity were equally highly enriched in molecular function, spectrin‐associated cytoskeleton was the most highly enriched in cellular component, and purine‐containing compound catabolic process was the most enriched in biological process (Fig. 2A). However, the threonine‐type peptidase activity and threonine‐type endopeptidase activity were equally highly enriched in molecular function; blood microparticles were the most highly enriched in cellular component; and negative regulation of multicellular organismal process was the most enriched in biological process in the GO functional clustering analysis of downregulated ubiquitinated sites with corresponding protein (Fig. 2B). Simultaneously, in the GO functional clustering analysis of all differentially expressed proteins, structural molecule activity was the most highly enriched in molecular function; extracellular space was the most highly enriched in cellular component, followed by blood microparticles; regulation of cell adhesion was the most highly enriched in biological process (Fig. 2C).

### *KEGG Pathway Analysis of Differentially Quantified Proteins*

KEGG is an information network that links known intermolecular interactions (http://www.kegg.jp/ or http://www.genome.jp/kegg/), as well as an encyclopedia of genes and genomes. The KEGG pathway mainly includes: metabolism, genetic information processing, environmental information processing, cellular processes, human diseases, drug development, and the like^39^.

**Figure 3:**
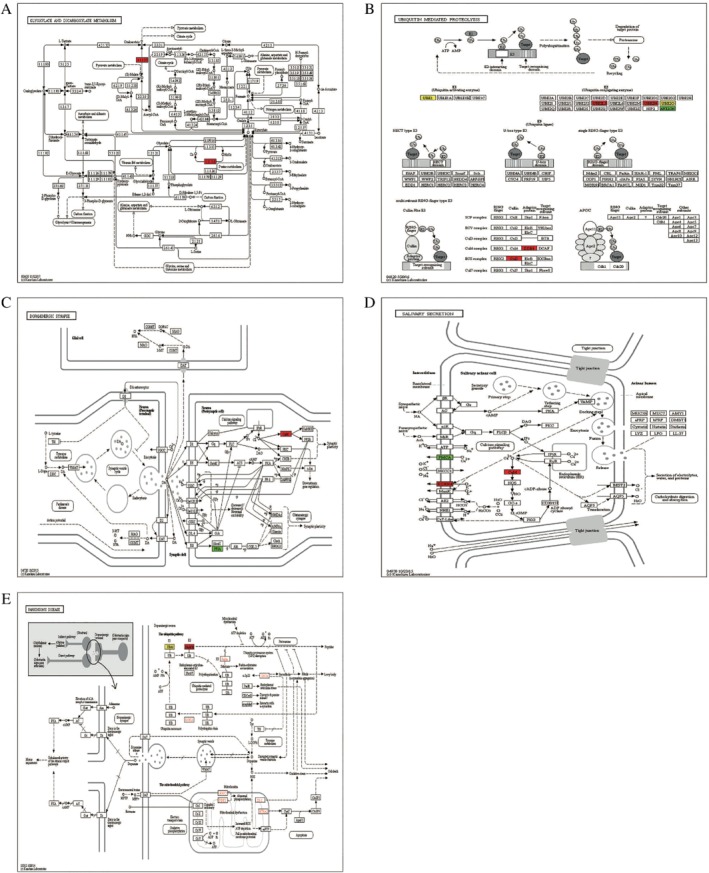
KEGG pathway of quantified proteins with upregulated ubiquitinated sites: (A) glyoxylate and decarboxylate metabolism; (B) ubiquitin mediated proteolysis; (C) dopaminergic synapse; (D) salivary secretion. (E) Parkinson's disease (red indicates the level of the protein is upregulated, bright green indicates the level of the protein is downregulated, and yellow indicates the presence of the node).

**Figure 4:**
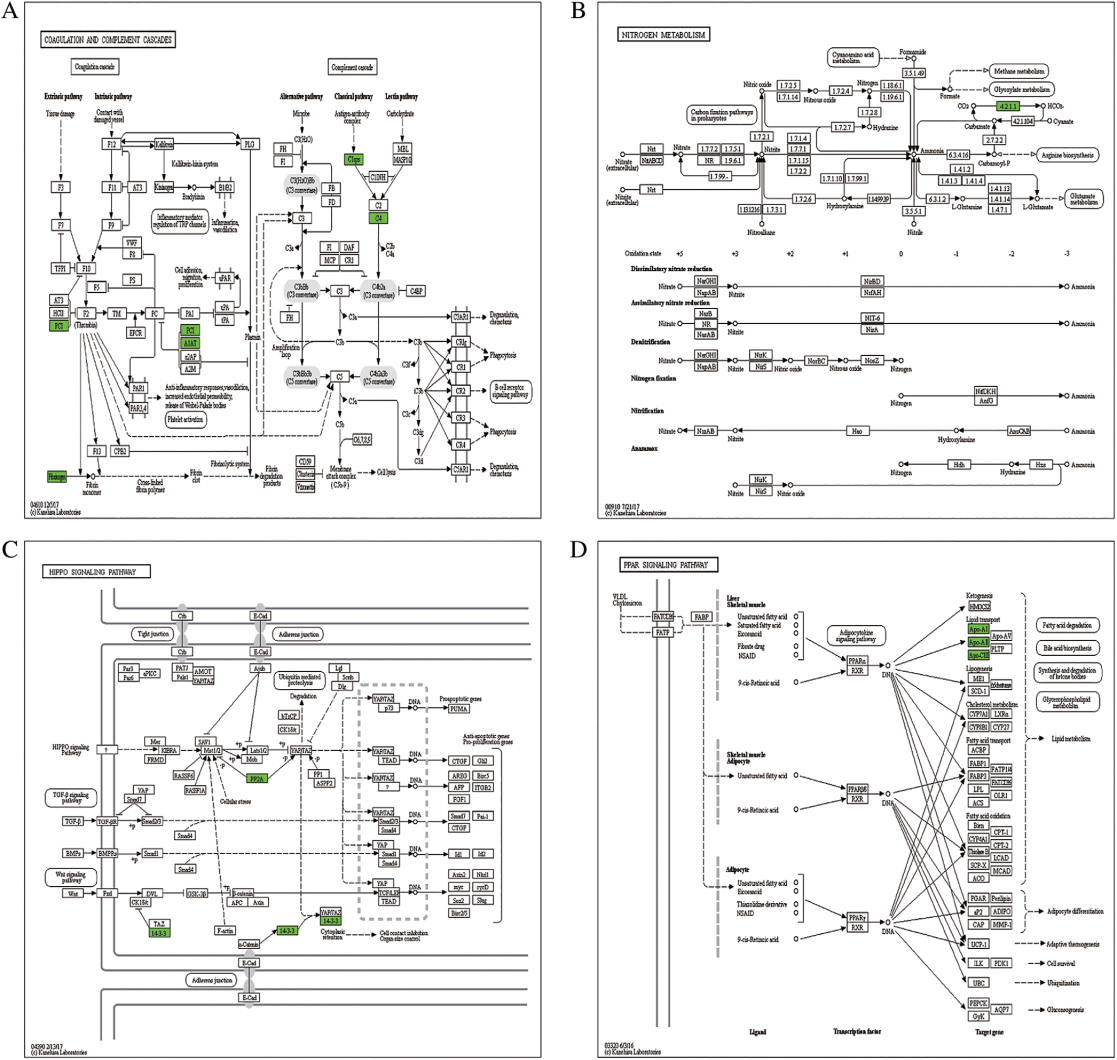
KEGG pathway of quantified proteins with downregulation ubiquitinated sites: (A) coagulation and complement cascades; (B) nitrogen metabolism (C) hippo signaling pathway (D) and the PPAR signaling pathway (green indicates the level of the protein is downregulated).

**Figure 5:**
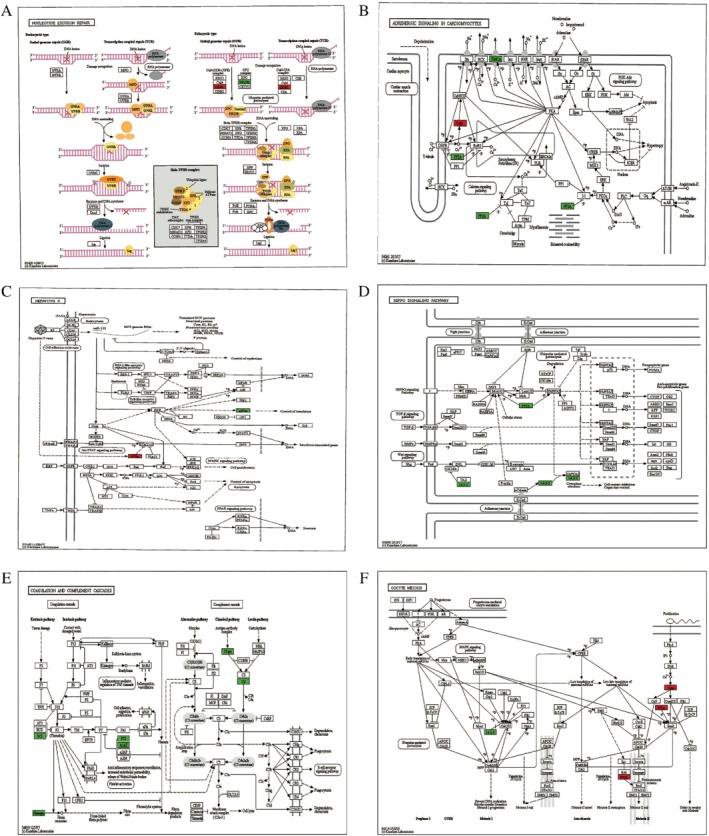
KEGG pathway of all quantified proteins: (A) nucleotide excision repair; (B) adrenergic signaling in cardiomyocytes; (C) hepatitis C; (D) hippo signaling pathway; (E) coagulation and complement cascades; and (F) oocyte meiosis (red indicates the level of the protein is upregulated, bright green indicates the level of the protein is downregulated, and yellow indicates the presence of the node).

The KEGG pathways of quantified proteins with upregulation ubiquitinated sites included glyoxylate and decarboxylate metabolism (Fig. 3A), ubiquitin mediated proteolysis (Fig. 3B), dopaminergic synapse (Fig. 3C), salivary secretion (Fig. 3D) and Parkinson's disease (Fig. 3E). However, the KEGG pathways of quantified proteins with downregulation ubiquitinated sites were coagulation and complement cascades (Fig. 4A), nitrogen metabolism (Fig. 4B), hippo signaling pathway (Fig. 4C), and the PPAR signaling pathway (Fig. 4D). The KEGG pathways of all quantified proteins were nucleotide excision repair (Fig. 5A), adrenergic signaling in cardiomyocytes (Fig. 5B), hepatitis C (Fig. 5C), hippo signaling pathway (Fig. 5D), coagulation and complement cascades (Fig. 5E), and oocyte meiosis (Fig. 5F).

**Figure 6:**
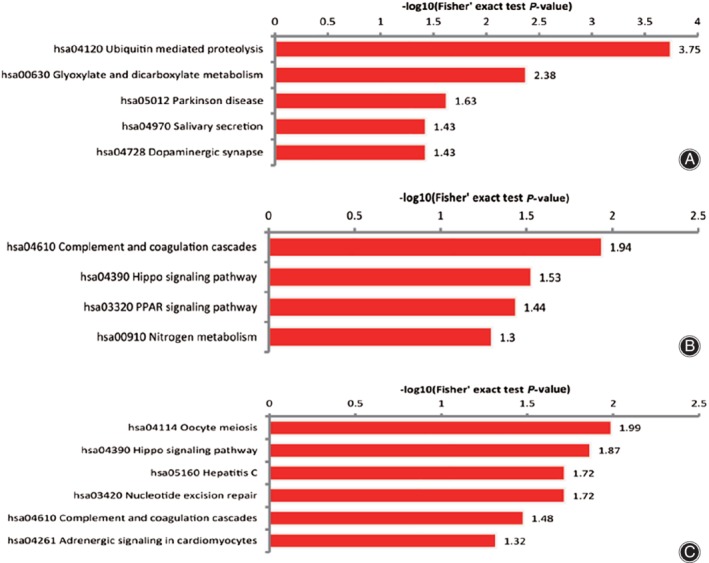
KEGG enrichment results of all quantified proteins: The horizontal axis value is a negative logarithmic transformation of significant *P*‐values (*P* < 0.05).

In the KEGG functional clustering analysis, hsa04120 ubiquitin‐mediated proteolysis was the most highly enriched in upregulated sites with corresponding proteins (Fig. 6A), hsa04610 complement and coagulation cascades were the most highly enriched in downregulated ubiquitinated sites with corresponding proteins (Fig. 6B), and hsa04114 oocyte meiosis was the most highly enriched in all differentially expressed proteins (Fig. 6C).

## Discussion

Quantitative analysis of ubiquitylomes was conducted in this study. The ubiquitylomes analysis of the whole blood in seven healthy postmenopausal women and seven postmenopausal osteoporosis patients demonstrated that 32 sites on 25 proteins were upregulated and 101 sites on 77 proteins were downregulated. However, increasing the number of samples may make the results more credible.

### *Gene Ontology Analysis of Differentially Quantified Proteins*

In our study, the GO analysis showed that cellular process, single‐organism process, biological regulation process, response to stimulus process, and metabolic process were leading biological process categories in quantified proteins both with upregulated ubiquitinated sites (Fig. 1A) and downregulation ubiquitinated sites (Fig. 1D). The top two functions of molecular function were binding and catalytic activity in quantified proteins with upregulated ubiquitinated sites (Fig. 1C) and downregulated ubiquitinated sites (Fig. 1F).

In contrast, the GO functional clustering analysis revealed that the enrichment results of the same cellular components, molecular function, and biological process vary widely between downregulated sites with corresponding proteins and upregulated sites with corresponding proteins.

Blood microparticles were the most highly enriched in cellular component of downregulated sites with corresponding proteins, and the second most highly enriched in cellular component of all differentially expressed proteins, it was not enriched in cellular component of upregulated sites with corresponding proteins. Ubiquitin conjugating enzyme activity and ubiquitin‐like protein conjugating enzyme activity were the most highly enriched in molecular function of upregulated sites with corresponding proteins, but they were not enriched in downregulated sites with corresponding proteins.

The above results suggest that there was a huge difference in cell biological activity between quantified proteins with upregulated ubiquitinated sites and downregulated ubiquitinated sites in postmenopausal osteoporosis.

### *KEGG Pathway Analysis of Quantified Proteins*

The KEGG pathway analysis of quantified proteins with differentiated ubiquitinated sites revealed 13 kinds of molecular interactions and functional pathways. Among them, glyoxylate and decarboxylate metabolism (Fig. 3A) and dopaminergic synapse (Fig. 3C) have both found in quantified proteins with upregulation ubiquitinated sites and neither found the relationship between them and bone metabolism. On the other hand, ubiquitin‐mediated proteolysis (Fig. 3B), salivary secretion (Fig. 3D), and Parkinson's disease (Fig. 3E) were reported all related to bone metabolism. These results reflect those of Fukushima (2017)^40^, who found that sustained osteoclast activity is largely due to accumulation of NOTCH2 carrying a truncated C terminus that escapes FBW7‐mediated ubiquitination and degradation. Salivary secretion may contribute to postmenopausal bone health. The most important clinically relevant finding was that MPTP‐induced Parkinsonian features in mice lead to trabecular bone loss through decreased bone formation and increased bone resorption due to changes in the serum circulating factors^41^, and Parkinson's disease was a risk factor for osteoporosis^42^.

Moreover, the hippo signaling pathway (Figs 4C and 5D), coagulation, and complement cascades (Figs 4A and 5E) were enriched both in all quantified proteins and the quantified proteins with downregulated ubiquitinated sites. These results indicated they were in significant position of bone metabolism. This also accords with the KEGG functional clustering analysis in downregulated ubiquitinated sites with corresponding proteins, which showed that hsa04610 complement and coagulation cascades are the most highly enriched (Fig. 6B).

However, the hippo pathway has been considered more important in previous studies of postmenopausal osteoporosis. In multicellular organisms, the hippo pathway is an identified signaling that plays an evolutionarily conserved fundamental role in organ size control, cell proliferation, cell apoptosis and fate determination of stem cells^42^, ^43^, ^44^. The hippo‐signaling pathway plays an important role in the osteogenic differentiation of mouse bone marrow mesenchymal stem cells^45^. Importantly, the hippo‐signaling pathway plays an important role in bone metastasis from breast carcinomar^45^.

The other molecular interactions and functional pathways of our study may also be involved in the regulation of bone metabolism in postmenopausal osteoporosis. More research is necessary to understand their roles in postmenopausal osteoporosis. This might help us comprehend the pathogenesis of postmenopausal osteoporosis, and the quantified proteins with differential regulation ubiquitinated sites may be potential diagnostic biomarkers in whole blood.

## *Conclusion*

The present study expands our understanding of the spectrum of novel targets that are differentially ubiquitinated in whole blood from healthy postmenopausal women and postmenopausal osteoporosis patients. Overall, these findings will contribute toward our understanding of the underlying proteostasis pathways in postmenopausal osteoporosis and the potential identification of diagnostic biomarkers in whole blood.

## Supporting information


**Table S1** The differentially expressed ubiquitinated sites and proteins in ubiquitylome of postmenopausal osteoporosis patients and healthy postmenopausal women.
